# Factors associated with health-related quality of life and burden on relatives of older people with multi-morbidity: a dyadic data study

**DOI:** 10.1186/s12877-020-01604-w

**Published:** 2020-06-26

**Authors:** Barbro Krevers, Anne Ekdahl, Tiny Jaarsma, Jeanette Eckerblad, Anna Milberg

**Affiliations:** 1 Department of Health, Medicine and Caring Sciences, Linköping University, Linköping, Sweden; 2 Department of Clinical Sciences Lund/Clinical Sciences Helsingborg, Lund University, Lund, Sweden; 3 Department of Neurobiology, Care Sciences and Society, Karolinska Institutet, Solna, Sweden

**Keywords:** Caregiving, Regression model, Evaluation, Cost effective, EQ5D index, COPE index

## Abstract

**Background:**

This study aimed to identify factors associated with health-related quality of life (HRQoL) and the burden on the relatives of older people with multi-morbidity.

**Methods:**

A secondary analysis of baseline data from 296 dyads, including older patients with multimorbidity and their relatives, which were previously collected in a randomized study.

The analysis was conducted to select correlated independent variables to enter a final linear regression analysis of two models with different endpoints: the relatives’ HRQoL (EQ5D index) and burden (COPE index: Negative impact scale).

**Results:**

Sixteen variables correlated with the relatives’ HRQoL, and 15 with the relatives’ burden. Both the HRQoL and burden correlated with both patient and relative variables. A high HRQoL was associated with relatives’ working/studying. A high burden was associated with caring for an older person with changed behaviour. A low burden was associated with the relatives’ high scores on positive values of caring, quality of support and HRQoL.

**Conclusion:**

Older persons and their relatives should be considered as a unit in the development of support of older people in order to increase the health and quality of life of both groups. To support and protect relatives from a high burden, potential measures could include improving the relative’s HRQoL and strengthening their ability to find positive values in care and strengthening reliable and good support from others. The relatives’ HRQoL explained the variation in the burden. However, the burden did not explain the variation in the HRQoL, which suggests that the relatives’ HRQoL is not so readily affected by their burden, whereas the relatives’ HRQoL can influence their burden. The variables used in the regression analyses where chosen to reflect important aspects of the relatives’ and older persons’ situations. The final models explained 38% of the variation in the relatives’ burden but only 10% of the variation in their HRQoL. This could be important to consider when choosing outcome assessments in future studies.

## Background

Relatives play an important role in the caring of older people. A consequence of the increase in the proportion of older people in the global population [1–3] is that an increasing number of relatives will be involved in care, involving a potential burden, as well as a negative impact on quality of life and health [4–8]. Much research has focused on informal carers for specific diagnostic and social groups, which may limit the generalizability and transferability of study results and conclusions regarding the common real-world situation of the high number of relatives of older people with multi-morbidity [9], of which many have multiple symptoms and frailty [10]. A recent scoping review of research on informal carers concluded that research on caring for people with multiple needs is still limited, and that the relationships between caring and carer outcomes are complex and not easy to assess properly. The authors call for an improvement to evaluation models and that more in-depth research is needed on the dyadic and relational situation for caring as it concerns and influences both the carer and the person being cared for [11]. For example, studies have shown that experience of minimal support from the family are associated with a low sense of security in patients [12], as well as their relatives’ perception of a high burden [13]. A previous study has indicated that an increase of the older person’s self-perceived health and psychological wellbeing were assoicated with relatives’ experience of care-related quality of life, which increased, and of their burden, which decreased [14]. Research based on dyadic data to investigate the situation of older persons and family carers is still rather scarce.

The question of how to properly assess the situation of the informal carer and the person being cared for concerns not only the design but also the choice of outcome variables that can reveal vital information about the dyadic situation. Burden is a frequently used outcome variable in studies on the relatives of older people. Although burden is an important aspect of caring, it only assesses the situation of relatives and we do not know how it refers to an older person’s situation. However, generic assessments of HRQoL, which are also frequently used, allow comparisons between different groups and interventions despite their different characteristics, and can also be used in cost-effective analyses. Thus, HRQoL has also been suggested as an outcome measure for informal carers in order to aggregate the effect on the HRQoL of both patients and relatives. However, these types of HRQoL assessments are constructed for evaluating interventions for patients. Thus, more research is required on generic HRQoL assessments when used on relatives [15]. We therefore wanted to know more about what influences the burden and HRQoL of the increasing number of relatives of older people with multi-morbidity, and how these two variables are interconnected. This would be helpful in using the outcome variables and in interpreting the evaluation results.

## Method

### Aim

Our aim was to identify factors associated with HRQoL and with the burden of relatives of older people with multi-morbidity.

### Design

This study is based on a secondary analysis of existing dyadic baseline data on older patients with multimorbidity and their relatives, previously collected in a randomized controlled study designed to evaluate an ambulatory geriatric unit. Two statistical regression models were developed to examine the explanatory factors for relatives’ perceived A) HRQoL and B) burden.

### Setting

Data were collected in a municipality in South-Eastern Sweden with a combined rural and urban population of approximately 130,000. Most health care was provided at 10 primary centres and one hospital with a total of around 300 beds, 12 specialist departments and 24-h admittance for surgical and medical emergencies. The municipality provided home health and social care when needed. In Sweden, county councils and municipalities are responsible for providing health and social care, funded mainly by income taxes.

### Participants

Inclusion criteria for older people with multi-morbidity were age ≥ 75 years, living in their regular home in a city in South-Eastern Sweden, having at least three concomitant medical diagnoses and having received inpatient hospital care at least three times in the previous 12 months. Out of a random sample of 844 eligible older people, 325 declined to participate, 79 had moved to an institution, 26 had died and 32 were unavailable. The remaining 382 older persons and 296 of their relatives took part in a baseline assessment. There were no additional inclusion or exclusion criteria for the relatives. Thus, the present analysis includes dyadic data from 296 older people with multi-morbidity and 296 relatives who were their closest caring relatives (Table 1).

**Table 1:** Characteristics of the participants: relatives (*N* = 296) and older people (*N* = 296)

Characteristics	Relatives		Older people	
	mean	sd	mean	sd
Age	66.7	13.8	82.5	4.7
	n	%	n	%
Female	198	66.9	139	47.0
Male	89	30.1	157	53.0
Relative’s relationship to the older person				
Spouse/partner (wife 73%, husband 27%)	140	48.3	–	–
Child (daughter 60%, son 40%)	132	45.5	–	–
Other	18	6.3	–	–
Single	41	14.3	136	46.3
Higher education (upper secondary school or university)	130	44.8	59	19.9
Main occupation ^a^				
Employed or self–employed work, studying	103	34.9	–	–
Retired (due to age)	178	60.3	–	–
Other	28	9.6	–	–
Relative cohabiting with older person	138	47.6	–	–
Older person living alone	–	–	135	45.9
Most common medical diagnosis of the older person ^b^				
Circulatory	–	–	284	95.9
Musculoskeletal	–	–	233	78.7
Psychiatric	–	–	102	34.5

Note: sd = standard deviation; *n* = number; % = valid percent

a. Multiple responses possible, b. All had at least three concomitant medical diagnoses

### Procedure

Older people who were potential participants were identified from a population-based administrative database maintained by the county council. All eligible participants received an invitation letter with information about the study and were then contacted by telephone. The older people who agreed to participate were asked to name their closest caring relative. These relatives also received an invitation and information letter. Independent collectors gathered baseline data following a structured protocol including asking the older people questions during a home visit. Relatives either completed a questionnaire during the baseline assessment of the older person (50%) or took part in a structured telephone interview (50%). The data collectors were trained care professionals but were not engaged in caring for the older participants. Both the older people and their participating relatives were informed of their right to withdraw from the study and were assured that their confidentiality would be maintained.

### Measures

To ensure coverage of important aspects of the caring situation, we consulted previous research to choose appropriate questions, instruments and scales. All the selected instruments and set of questions have been used in other studies of older people and relatives, in both Sweden and in other countries, and have demonstrated proper validity and reliability.

### Data and assessments reported by relatives

#### Demography

 Age, sex, civil status, educational level, occupation.

#### Health-related quality of life (HRQoL)

-EQ-5D index (Dependent variable in regression model A): Self-reported health status in five domains (mobility, self-care, usual activities, pain/discomfort, anxiety/depression with three-level response scales) and transformed into an index based on the public’s valuation of quality of life related to different health states (−.594 = worse than dead to 1 = full health) [16].

#### Burden

-Negative impact scale in COPE index (Dependent variable in regression model B): A summary of seven questions on: emotional well-being, physical health, overly demanding caring, difficulties in relationships with family, with friends, feeling trapped, financial difficulties (4-point Likert scale 1 = never to 4 = always, total scale 7 = never to 28 = always) [17].

-Hours of care per week provided by the relative of the older person.

#### Coping

-Positive value in caring: Positive value scale (4 item) in COPE index: questions on coping with caring, finding caring worthwhile, experiencing a good relationship with the person, cared for, and being appreciated for providing care (4-point Likert scale: 1 = never to 4 = always, total scale 4 = never to 16 = always) [17].

-Attachment security profile: Experiences in close relationships (ECR-16) contains two subscales: Anxiety scale, a measure of fear of rejection and abandonment (8 item) and Avoidance scale, a measure of discomfort with closeness and dependence on close others (8 item), (7-point Likert scale: 1 = disagree to 7 = agree, higher scores on each scale indicate greater attachment insecurity) [18].

#### Relatives’ sense of security and support

-Sense of security in care – Relatives’ evaluation (SEC-R) contains threes subscales: Care interaction scale (7 item), Mastery scale (5 item), Patient situation scale (5 item) (6-point Likert scale:1 = never to 6 = always) [19].

-Quality of support scale (4 item) in COPE index: questions on support received from friends and neighbours, from family, and from health and social services, and perceived overall support (4-point Likert scale: 1 = never to 4 = always, total scale 4 = never to 16 = always) [17].

#### Relatives’ perceptions of the older person’s health

-Relatives’ perceptions of weather the older person has changed behaviour that affects the interaction with the relatives and make them sad or upset: a single question from the EUROFAMCARE Comprehensive Assessment Tool (5-point Likert scale: 1 = never to 5 = very often) [20], based on a modified Behavioural and Instrumental Stressors in Dementia (BISID) [21].

-Relatives’ expectation of the older person’s future health development over the next year: a single question from the Patient Perspective on Care and Rehabilitation Process instrument (POCR) (1 = recovered, 2 = neither good nor poor, 3 = deteriorating) [22].

### Data and assessments reported by the older person

#### Psychological health

Geriatric Depression Scale 15-item (GDS) (yes/no, total score 1 = not depressed to 15 = depressed) [23].

#### Older person’s sense of security and support

-Sense of security in care – Patient’ evaluation (SEC-P) contains threes subscales: Care interaction scale (8 item); Identity scale (4 item), Mastery scale (3 item), (6-point Likert scale: 1 = never to 6 = always) [24].

-Quality of support scale (4 item) in COPE index: questions on support received from friends and neighbours, from family, and from health and social services, and perceived overall support (4-point Likert scale: 1 = never to 4 = always, total scale: 4 = never to 16 = always) [17].

### Data analysis

We conducted uni-, bi- and multivariate analyses, including a commonality analysis, to select unique impacts of correlated independent variables to enter in a final linear regression analysis of two models. The EQ5D index was the dependent variable in model A (HRQoL) and the negative impact scale of the COPE index was the dependent variable in model B (burden). We used a Pearson’s correlation in the parametric bivariate analysis for the continuous variables with normal distribution and a non-parametric Spearman’s Rho correlation analysis for the categorical data. Independent variables were selected for use in an initial forward stepwise regression (Table 2). Variables with an alpha level of < 0.09 were included, and an alpha level 0.10 was set as a limit for removal. We used listwise exclusion to handle internal missing values. Stepwise regression was conducted to select variables for the final two ENTER regression models. In the final models, three independent variables were used in model A and four were used in model B (Table 3). The alpha level was set to 0.05 for significance in both models.

**Table 2:** Relatives’ HRQoL (EQ5D index) and burden (Negative impact scale in COPE index) and their bivariate associations with the selected variables

Variables	HRQoL			Burden		
Relatives’ data	r	rho	*p*	r	rho	*p*
***Demography***						
Spouse/partner (0 = no; 1 = yes)		−.16	.007*		−.12	.042*
Work/studying (0 = no; 1 = yes)		.28	<.001*		.01	.892
Education level (0 = none to 4 = high/university level)		.18	.003*		.16	.009*
***HRQoL*** (−.594 = worse than dead to 1 = full health)				−.182		.003*
***Burden and strain***						
Burden (7 = never to 28 = always)	−.182		.003*			
Care hours/week provided by the relative		−.16	.017*		.20	.002*
***Coping***						
Positive value in caring in COPE index (4 = never to 16 = always)	.042		.483	−.493		<.001**
Anxiety in close relationships (1 = low to 7 = high)	−.190		.002*	.080		.188
Avoidance in close relationships (1 = low to 7 = high)	−.125		.040*	−.024		.701
***Sense of security and support***						
Care interaction (1 = never to 6 = always)	.133		.038*	−.225		<.001**
Mastery (1 = never to 6 = always)	.132		.027*	−.360		<.001**
Patient situation (1 = never to 6 = always)	.127		.039*	−.230		<.001**
Quality of support (4 = never to 16 = always)	.030		.62	−.395		<.001**
***Older person’s health***						
Changed behaviour (1 = never to 5 = very often)	−.215		<.001*	.474		<.001**
Perception of future health trajectory (1 = recovered; 2 = neither good nor poor; 3 = deteriorating)		−.13	.024*		.26	<.001**
**Older persons’ data**						
***Health***						
General depression (1 = not depressed to 15 = depressed)	−.160		.007*	.197		.001**
***Sense of security and support***						
Care interaction (1 = never to 6 = always)	.124		.038*	−.084		.16
Identity (1 = never to 6 = always)	.093		.118	−.150		.012*
Mastery (1 = never to 6 = always)	.116		.050*	−.183		.003*
Quality of support (4 = never to 16 = always)	.162		.006*	−.138		.022*

Bold figures = variables used in the stepwise regression analysis

*p ≤ 0.05; ** ≤ 0.001

**Table 3:** Linear regression models showing the independent variables that best explain the variation in HRQoL (EQ5D index) and burden (Negative impact scale COPE index)

**MODEL A: Dependent variable**	**HRQoL** (*n* = 227)						
	B	95% CI for B		β	*p*	R^2^	Adj R^2^
Independent variables		Lower	Upper				
Relatives working/studying (0 = no;1 = yes)	.139	.074	.204	.275	<.001*		
Care interaction perceived by relatives (1 = never to 6 = always)	.027	−.004	.057	.117	.086		
Quality of support perceived by older people (4 = never to 16 = always)	.011	−.002	.023	.112	.087		
Burden perceived by relatives (7 = never to 28 = always)	−.007	−.016	.001	−.111	.093		
Explained variance						.112	.096
**MODEL B: Dependent variable**	**Burden** (*n* = 255)						
	B	95% CI for B		β	*p*	R^2^	Adj R^2^
Independent variables		Lower	Upper				
Relatives’ health–related quality of life (−0.594 = worse than death to 1 = full health)	−1.732	−3.272	−.193	−.112	.028*		
Positive value in caring perceived by relatives (4 = never to 16 = always)	−.468	−.646	−.290	−.307	<.001*		
Quality of support perceived by relatives (4 = never to 16 = always)	−.211	−.352	−.070	−.171	.003*		
Changed behaviour of older person perceived by relatives (1 = never to 5 = very often)	1.002	.660	1.344	.308	<.001**		
Explained variance						.386	.376

Unstandardized coefficient (B) with 95% confidence interval (CI), standardized coefficient (β) for the independent variables, coefficient of determination (R^2^) for overall model fit, adjusted coefficient of determination (adj R^2^) regarding multiple independent variables

*p ≤ 0.05; ** ≤ 0.001

## Results

Ages ranged from 31 to 91 years in the relatives and from 75 to 96 years in the older people. Table 1 contains further information about the participants.

### Correlation analysis

There was a significant relationship (*p* < 0.05) between the two concepts under investigation and other variables. The HRQoL correlated with 16 out of 19 variables and burden with 15 out of 19 variables (Table 2). The relatives’ HRQoL was negatively correlated with burden, and vice versa. Four out of five variables that derived from the older persons’ reported data such as general depression, their sense of security and quality of support, were positively correlated with relatives’ HRQoL and negatively correlated with relatives' burden. The strongest correlations for each endpoint are summarized below.

The correlations indicate that the HRQoL was higher in relatives who were working or studying, had a higher education, and when the older person perceived a higher quality of support, while the HRQoL was lower in relatives caring for an older person whose behaviour had changed, those who showed higher anxiety in close relationships in their attachment profile and those who perceived a higher burden. Relatives’ perceptions of a lower burden correlated with situations in which relatives perceived higher positive values in caring, higher quality of support and higher sense of mastery over the care situation, whereas, relatives’ perceptions of a higher burden were associated with situations in which the older persons had changed their behaviour, relatives expected the older person’s health to deteriorate and relatives provided a higher amount of care hours per week (Table 2).

### Regression analysis

The final regression model A with the HRQoL as a dependent variable included four independent variables, of which only working or studying were statistically significant. Selected variables, though not significant, were relatives’ burden, their perceived sense of security in care interaction and the quality of support perceived by older people. Not working or studying were associated with a decreased HRQoL (EQ5D index) by 0.139 on the scale. This variable had a direct influence of 28% on the variance of HRQoL and the whole model explained 10% of the variance in HRQoL when adjusted for multiple variables (Table 3).

The final regression model B with burden as a dependent variable included four independent variables. The result showed that an improvement of 0.10 on the EQ5D index of relatives’ HRQoL was associated with a decreased burden of 1.70 scale steps. Furthermore, 1-step increases on the scale of relatives’ positive value of caring and quality of support were associated with a decrease in burden by 0.47 and 0.21 scale steps, respectively. As an older person’s behaviour deteriorated by one step on this scale, the relatives' burden increased by around one on the negative impact scale. The direct influence on the variance of the burden was as follows: changed behaviour in the older person (31%), positive value in caring (31%), quality of support (17%) and relatives’ HRQoL (11%). When adjusted for multiple variables, the whole model explained 38% of the variance in the burden (Table 3).

## Discussion

Our study on dyadic data offers in-depth information about factors connected to the HRQoL and burden in relatives of older people with multi-morbidity. It also provides valuable information about the association between these two outcome variables, investigated through two regression models A (HRQoL) and B (burden). Most of the examined variables have significant bivariate associations with HRQoL (16 out of 19) and burden (15 out of 19), and some of them demonstrate the dyadic aspect of caring, such as the older people’s sense of security, perceived quality of support, and mental health. Of the four selected variables in model A, only one – working/studying – explained the variation in relatives’ HRQoL, whereas, all four of the four selected variables in model B explained variations in the relatives’ burden: relatives’ perceptions of changed behaviour in the older person, positive value in caring, quality of support and the relatives’ HRQoL.

The results indicate that the relatives’ involvement in work or study is associated with a higher HRQoL. Other researchers have found that relatives who can balance work and family life with a caring situation also report higher levels of life satisfaction and well-being [25]. Having a job may also improve a relative’s health, opportunities for social contact and their financial situation [9, 26]. However, if the family caring role involves a high burden, it may be difficult to achieve this balance, which may lead to the person leaving their work [27, 28]. Our results indicate that relatives who are working or studying probably represent a younger generation and also a healthier cohort with better physical and psychological resilience than the total sample of relatives. Their situation as caring relatives has not noticeably influenced their health or their HRQoL. Another reason could be that in Sweden, the younger generation seldom cohabit with the older generation and do not have to deal with the needs of older persons in everyday activities. A probable prerequisite is that the working group of relatives also perceive that an adequate level of health and social care is being provided for the older person who, in this case, is probably a parent living alone. There are indications that older people who are single receive more home services than cohabiting spouses [29].

The four explanatory factors of burden demonstrate that the relatives’ caring situation is embedded in their total life situation, which also embraces the older persons’ situation. The relatives’ HRQoL is one of the explanatory factors of burden and it shows an association pattern of higher burden – lower HRQoL, and vice versa. This is a pattern that has been described in other studies on relatives of older people [4, 5, 30]. However, our study also demonstrates that the burden did not explain the change in HRQoL (regression model A). These findings suggest that relatives with a higher HRQoL can handle even a strained caring situation, while a lower HRQoL will have a negative effect on the relatives’ perceived burden. Relatives in poor health and with a correspondingly low HRQoL will find it difficult to handle their own situation while also providing support to an older person who is unwell. Some relatives, often spouses, also have multiple-chronic conditions, which impact their ability to care [31].

In our results, caring for an older person with changed behaviour is related to higher burden. This result is in line with other studies on the relatives of persons with cognitive impairment, for example, from dementia or stroke [13, 32–34]. In our sample, older people with multi-morbidity had at least three concomitant medical diagnoses and had received inpatient hospital care at least three times in the previous 12 months, implying they are all potentially frail. When changed behaviour is included in this frailty, the results suggest that relatives feel an increased burden. Their interaction with the older person, including relationships and roles, might have changed, which can cause a sense of loss and frustration [35].

When considering ways of improving a relative’s situation, there are important findings to consider, such as the relationship between a lower burden and higher scores on relatives’ positive value in caring and on quality of support. Strengthening family carers’ perceptions of positive values as a coping strategy to decrease their burden and strain has been suggested in other studies [17, 19, 36]. Finding positive values in caring may have a protective effect against the potential negative impact of caring. Although this could be a “chicken and egg” situation, there are good arguments for the importance of this factor and its inclusion in designing support for relatives. In our assessment, the positive value scale in the COPE index was used, which includes questions on coping with caring, finding caring worthwhile, having a good relationship with the person being cared for, and being appreciated for providing care. It demonstrates that positive value includes aspects of interaction with others. Thus, the relatives’ perception of positive value in caring could be related to their perception of the quality of support which, in our assessment, includes support from family, friends, health and social care, as well as overall support. Such support has been shown to be very important for relatives [4, 9, 13].

### Considerations

There are some circumstances that may have implications for the interpretations of the results. This study drew on a randomized sample of older people. In order to obtain paired data, only those who were dyads, i.e. older persons who had a participating relative, could be included. However, the relatives in our sample were demographically similar to those in other Swedish studies of the relatives of older people [37] and, like those studies, represented different situations. The varied sample probably contributed to less statistically significant results, although it does reflect a real-world situation. When interpreting the results, it is important to remember that the HRQoL was measured on the EQ-5D index, which assesses five domains of HRQoL related to symptoms and abilities. The EQ-5D index provides a societal perspective as it reflects the public values of different reported health states in relation to HRQoL. An advantage of the measure is that it avoids individual coping, for example, that over time, people adapt to more severe health problems. A disadvantage could be that it does not show the relatives’ own values. Furthermore, the analysis involved multiple testing, which may cause a type I error (reject a true null hypothesis). However, our analysis was exploratory (rather than testing null hypothesis), based on regression model selection techniques in which the p-values support the interpretation. Finally, the study was cross-sectional, so the results should be interpreted with caution, the observed associations among variables describe a relationship but not the causal direction.

## Conclusions

The results indicate that older persons and relatives should be considered as a unit in the development of support of older people in order to increase health and quality of life for both, as well as in evaluations. In particular, relatives of older people with multi-morbidity and a deteriorating mental state (depression and changed behaviour) and relatives with a lower HRQoL are at risk of a high burden. In order to support and protect relatives from a high burden, potential measures could be improving relatives’ HRQoL and strengthening their ability to find positive values in care, strengthening reliable and good support from others, as well as strengthening support for the older person. The results call for further investigation of the interplay between the situation of elderly persons and their caring relatives, through dyadic studies.

The regression models explained more of the variation in the burden (38%) than in the HRQoL (10%). Consequently, there are aspects other than the variables that were selected in the present analysis that influence a relatives’ situation, particularly their HRQoL. Furthermore, the relatives’ HRQoL explained the variation in the burden, although the burden did not explain the variation in the HRQoL. These findings suggest that the relatives’ HRQoL is not so easily affected by their burden, whereas the relatives’ HRQoL can influence their perceived burden. Our study also highlights the risk that assessments of HRQoL (EQ5D index) can underestimate the effect of the caring situation, particularly on younger carers (who are working), because young people generally have better health that can mask the effects more readily seen in older carers. This highlights the need for HRQoL instruments that also capture social interaction as this has shown to be an important aspect of being a relative caring for a family member, as well as being an older person in need of support from others. These results should be considered when deciding on interventions and choosing outcome assessments in future studies on the relatives’ of older people with multi-morbidity.

## Data Availability

The datasets generated and/or analysed during the current study are not publicly available due to Swedish legislation on ethics in research but are available from the corresponding author.

## Abbreviation

HRQoL: Health-related quality of life
